# Hypothyroidism Due to Seaweed Overconsumption

**DOI:** 10.7759/cureus.55231

**Published:** 2024-02-29

**Authors:** Kazuki Unosawa, Tetsuro Aita, Sugihiro Hamaguchi

**Affiliations:** 1 Department of General Internal Medicine, Fukushima Medical University, Fukushima, JPN; 2 Department of Internal Medicine, Fujita General Hospital, Kunimi, JPN

**Keywords:** wolff–chaikoff effect, skin rash, thyroid autoantibodies, excessive iodine intake, primary hypothyroidism

## Abstract

Hypothyroidism presents various symptoms, ranging from commonly observed signs, such as fatigue, cold sensation, and constipation, to rare features, such as rash and pancytopenia, resembling certain rheumatological and hematological diseases. Chronic, excessive iodine consumption causes primary hypothyroidism. However, when iodine overconsumption becomes a regular part of daily dietary habits, it becomes difficult for patients to associate their symptoms with daily iodine consumption. Therefore, clinicians cannot obtain information on excessive iodine intake from the patient. Here, we present a case of hypothyroidism that was subsequently identified as caused by excessive dairy seaweed consumption for health purposes. This case report highlights the importance of a detailed dietary history in patients diagnosed with primary hypothyroidism without thyroid autoantibodies.

## Introduction

Hypothyroidism results from a deficiency in the thyroid gland and commonly presents with symptoms such as fatigue, lethargy, constipation, dry skin, and weight gain. Although relatively rare, hematological and cutaneous abnormalities can occur [1]. Diagnosis is primarily based on biochemical results because the disease usually lacks specific symptoms. Iodine is necessary for thyroid hormone synthesis, and exposure to excess iodine results in abnormal thyroid function (hyperthyroidism and hypothyroidism) [2]. Because iodine is mainly taken in through food, prolonged consumption of excessive amounts of iodine without realizing it can lead to hypothyroidism. The primary route of excessive iodine intake is the overconsumption of iodized salt, iodine-rich milk, seaweed, iodine-containing drinking water, and iodine-containing dietary supplements [3]. However, it may be challenging for clinicians to identify hypothyroidism caused by the daily consumption of iodine-containing foods without a detailed dietary history. Additionally, patients may not associate their symptoms with excessive seaweed consumption unless they are specifically asked about them. In this way, when excessive iodine intake becomes part of one's diet, both clinicians and patients may not easily recognize it as a cause of hypothyroidism. Therefore, when encountering cases of hypothyroidism with unknown causes, it is crucial to take a detailed dietary history.

In this report, we present a case of hypothyroidism caused by chronic overconsumption of seaweed for health purposes. The patient was diagnosed through a detailed food consumption history triggered by abnormalities in the fecal contents found on an abdominal X-ray.

## Case presentation

A 43-year-old man presenting with weakness, constipation, dry skin, and cellulitis was referred to our hospital. He was previously a healthy science teacher at a high school and had been in his usual state of health until two years earlier when he was found to have anemia and leukopenia during an annual health checkup. Bone marrow examination revealed atypical red blood cells without evidence of chromosomal abnormalities, leading to a provisional diagnosis of low-risk myelodysplastic syndrome (MDS). A short course of azacitidine therapy did not achieve hematological recovery; therefore, the patient was managed without chemotherapy. One year before referral, he developed a painful swelling in his left foot and was treated for cellulitis with antibiotics at a local clinic. Six months earlier, he began experiencing constipation, cold sensations, and skin roughness or cracking of the extremities. Muscle weakness gradually progressed over the past three months. He developed painful swelling below the left knee 10 days prior, prompting a visit to the hospital where he was being followed up for MDS. He had dry skin, fissures, and skin cracking on his hands. Laboratory tests revealed anemia, leukocytopenia, and liver enzyme abnormalities. The levels of thyroid-stimulating hormone and free thyroxine were 36.3 μIU/mL and <0.40 ng/mL, respectively. As he had difficulty moving because of weakness and malaise, he was hospitalized with a diagnosis of cellulitis and hypothyroidism. Cefazolin was administered for the cellulitis, and thyroid hormone supplementation was initiated. Due to concerns about the skin on his hands resembling “mechanic’s hands” and the possibility of an underlying process such as dermatomyositis or anti-synthetase syndrome, he was transferred to our hospital for further evaluation and management.

**Figure 1:**
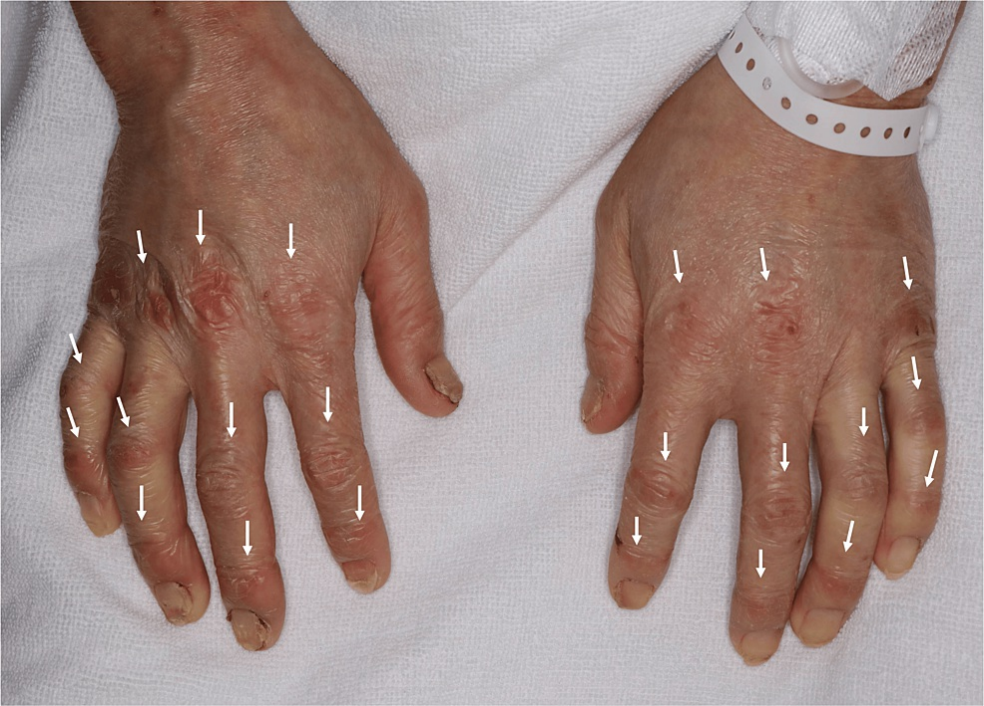
Skin rash on the hands and finger joints Skin rash on the dorsal aspects of distal and proximal interphalangeal joints and metacarpophalangeal joints, which resembled Gottron's sign or mechanic's hands, typically seen in patients with dermatomyositis or anti-synthetase syndrome.

On admission to our hospital, blood pressure was 97/60 mmHg, pulse 70 bpm, body temperature 35.6°C, respiratory rate 18 breaths/min, and oxygen saturation 98% on ambient air. Alopecia, dry skin, and chilblain-like skin rashes on his ears, elbows, fingers, and feet were noted. The skin rash on the hands and finger joints resembled Gottron’s sign or mechanic’s hands (Figure 1). No goiter was noted. The abdomen was distended due to diminished bowel sounds. A swollen erythematous lesion suggestive of a subcutaneous abscess was observed below the left knee. Mild generalized muscle weakness was also observed.

Further laboratory investigations revealed that anti-nuclear, anti-double-stranded DNA, anti-aminoacyl-tRNA synthetase, anti-RNA polymerase III, and anti-SSA/SSB antibodies were all negative. Although the patient had hypothyroidism, both anti-thyroid peroxidase and anti-thyroglobulin antibodies were negative. Culture of the aspirated fluid from the subcutaneous abscess revealed methicillin-sensitive *Staphylococcus aureus*, and cefazolin was continued.

**Figure 2:**
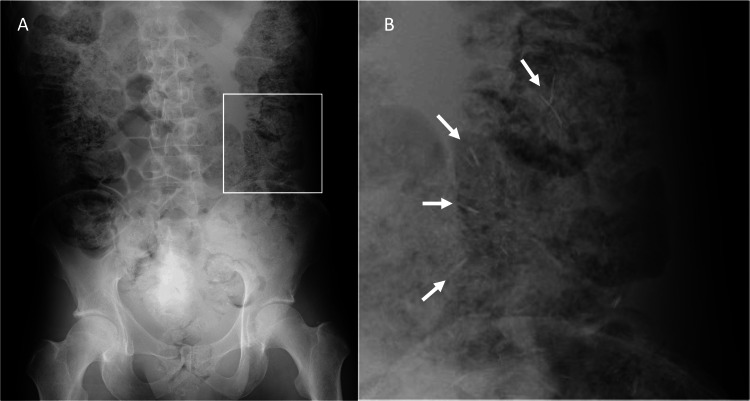
Abdominal X-ray (A) Prominent gas and a large amount of feces in the entire colon. (B) Enlarged view of the white square area of (A). A large amount of thin fibrous materials present within the feces.

**Figure 3:**
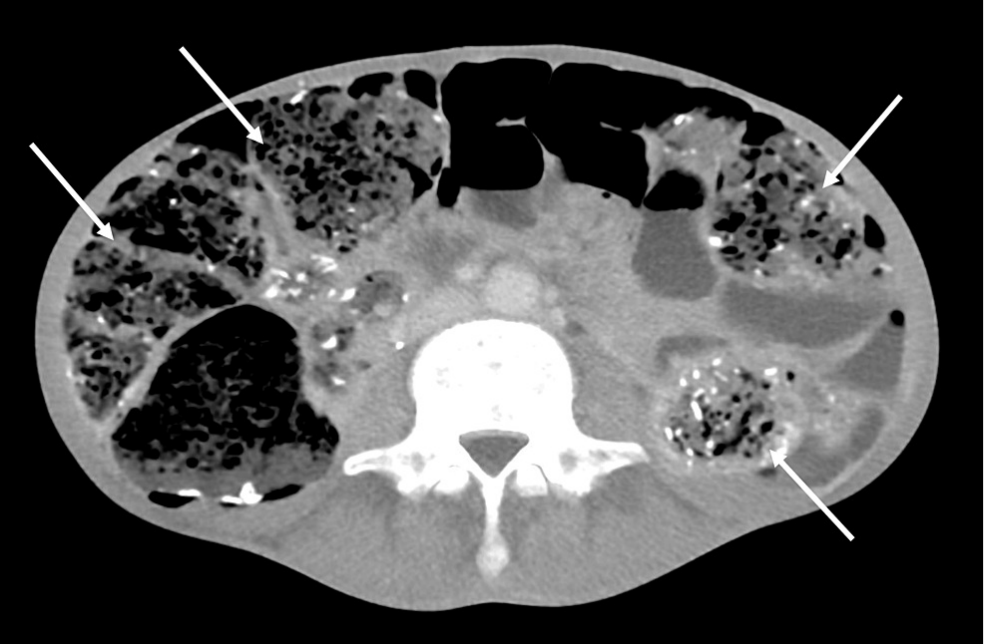
Computed tomography of the abdomen Dilated colon filled with a large amount of feces containing gas and some high-density materials.

An abdominal X-ray revealed prominent gas and feces in the colon, with a large amount of thin fibrous material within the feces (Figure 2). Computed tomography showed no abnormalities in the thyroid gland; however, the colon was dilated and filled with a substantial quantity of feces containing gas and high-density materials (Figure 3).

We took the patient’s dietary history again and found that the patient began consuming tororo kombu (shredded kelp) during meals for health purposes three years earlier. Constipation developed approximately two years earlier, leading to an increased daily intake of tororo kombu as it was believed to be effective for constipation. Over the previous six months, the patient had been consuming tororo kombu at every meal, resulting in an estimated iodine intake of 40-80 mg per day, far exceeding the recommended daily intake of iodine for adults in Japan, which is 0.13-0.15 mg. The diagnosis of hypothyroidism due to excessive seaweed consumption was confirmed. Thyroid hormone replacement therapy (THRT) was initiated.

**Figure 4:**
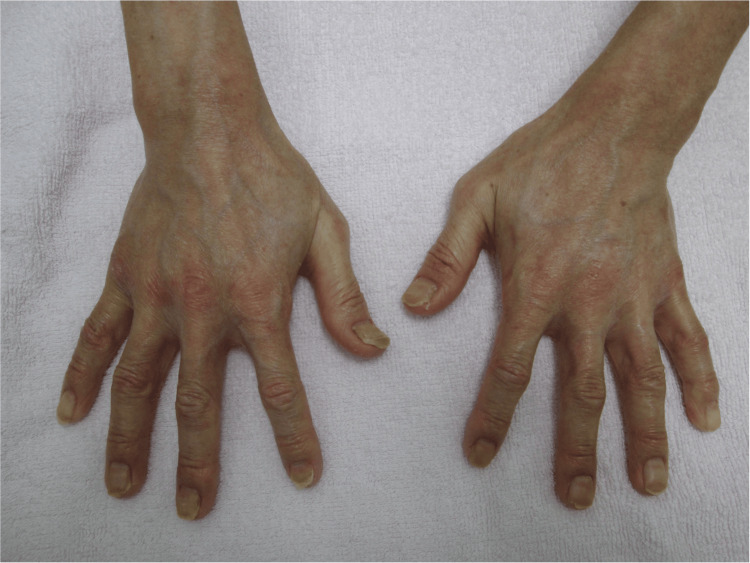
Hands after one month of thyroid hormone replacement therapy Skin lesions observed at the time of admission improved after thyroid hormone levels became normal.

With approximately one month of THRT, his thyroid function normalized, and the skin lesions, including rashes on the hands and finger joints, improved (Figure 4). 

He was discharged home with instructions to limit intake of iodine-containing foods such as tororo kombu. As his thyroid function normalized one month after discharge, THRT was discontinued. In addition to the improvement in symptoms and other abnormal findings that were present at the time of admission to our hospital, blood cell abnormalities such as anemia and leukopenia also improved six months after discharge. He subsequently returned to work as a science teacher, and his thyroid function remained normal without the need for THRT for the following two years.

## Discussion

We report the case of a previously healthy middle-aged man who developed hypothyroidism due to chronic excessive iodine intake for health benefits. His manifestations resembled those of collagen vascular diseases; however, the diagnosis was successfully made after taking a detailed food consumption history based on the abnormalities detected on the abdominal X-ray.

Excessive iodide administration can transiently reduce the production of thyroid hormones, a phenomenon known as the Wolff-Chaikoff effect [4]. Normal thyroid hormone synthesis typically resumes within 24 h of adaptation, even with continuous iodine administration [5]. However, failure to adapt to the Wolff-Chaikoff effect can occur in susceptible patients with underlying risk factors such as autoimmune thyroid disease, subacute thyroiditis, postpartum thyroiditis, type 2 amiodarone-induced thyrotoxicosis, interferon-alpha therapy, concomitant use of other potential goitrogens (e.g., lithium), and Graves’ disease treated with antithyroid drugs, thyroidectomy, or radioactive iodine [2]. In our case, adaptation after excessive iodide intake was unlikely even in the absence of underlying risk factors, and the patient’s hypothyroidism persisted. This was likely due to the patient’s habit of consuming large amounts of iodine-rich foods daily for a prolonged period. A study examining the association between dietary iodine intake and the prevalence of autoantibody-negative hypothyroidism indicated that, in iodine-sufficient areas, the prevalence of hypothyroidism was higher in individuals with high iodine intake than in those with low iodine intake. In addition, hypothyroidism is more frequent and severe in individuals who consume excessive quantities [6]. He also recovered completely and did not develop permanent hypothyroidism after discontinuation of THRT. This indicates that the patient did not have any underlying risk factors for thyroid dysfunction.

Hematological abnormalities, especially anemia, have been reported in approximately 30% of patients with hypothyroidism [7]. Severe hypothyroidism can lead to pancytopenia. Our patient was diagnosed with MDS two years earlier, but the pancytopenia improved after THRT. Therefore, we are confident that the cause of pancytopenia at that time was hypothyroidism rather than MDS. Autoimmune processes associated with thyroid autoantibodies, such as pernicious anemia or direct autoimmune reactions to the bone marrow, may cause marrow hypoplasia [8]. However, thyroid autoantibodies were negative in our case. A case of severe hypothyroidism without autoantibodies in a patient who developed pancytopenia has been reported, indicating that hypothyroidism itself can cause pancytopenia regardless of the autoimmune process [9]. Recovery from pancytopenia varies according to reports, ranging from one week to two months with thyroid hormone supplementation [10].

Patients with hypothyroidism may present with various cutaneous manifestations, including xerosis, diffuse hair loss, melasma, chronic urticaria, generalized pruritus, tinea corporis, alopecia areata, vitiligo, lichen planus, and xanthelasma palpebrarum [11]. It is also associated with a thick, dry, cold, and pale skin. Keratoderma is often observed on hands and feet [12]. In our case, the dry and thickened skin with keratoderma mimicked Gottron’s sign or mechanic’s hands, suggesting possible underlying collagen vascular diseases.

There is a cultural habit of consuming seaweed for its perceived health benefits, particularly in Asian countries such as Japan and Korea [13]. The upper limit for iodine intake in healthy adults varies by country, with the World Health Organization recommending 0.15 mg per day [14], while in Japan, it is set at 3 mg per day [15]. However, in the present case, the patient’s daily iodine intake significantly exceeded these levels. Since the consumption of tororo kombu had become part of the patient’s diet, we initially could not associate it with his symptoms. Our case provides valuable information on the importance of obtaining a detailed dietary history in patients diagnosed with hypothyroidism without thyroid autoantibodies.

## Conclusions

Chronic excessive iodine consumption is one of the causes of primary hypothyroidism. Herein, we present the case of a patient who developed hypothyroidism due to excessive long-term iodine intake for perceived health benefits. Initially, we could not obtain information about this dietary habit from the patient and thus did not associate his symptoms with regular seaweed consumption. This case highlights the significance of obtaining a comprehensive dietary history in patients diagnosed with hypothyroidism without thyroid autoantibodies.
